# A Systematic Review and Meta-Analysis on the Effects of Probiotic Species on Iron Absorption and Iron Status

**DOI:** 10.3390/nu11122938

**Published:** 2019-12-03

**Authors:** Susan C. Vonderheid, Lisa Tussing-Humphreys, Chang Park, Heather Pauls, Nefertiti OjiNjideka Hemphill, Bazil LaBomascus, Andrew McLeod, Mary Dawn Koenig

**Affiliations:** 1 College of Nursing, University of Illinois at Chicago, Chicago, IL 60612, USA; vonde@uic.edu (S.C.V.); parkcg@uic.edu (C.P.); hpauls2@uic.edu (H.P.); 2 Department of Medicine and Cancer Center, University of Illinois at Chicago, Chicago, IL 60608, USA; ltussing@uic.edu; 3 Department of Kinesiology and Nutrition, University of Illinois at Chicago, Chicago, IL 60612, USA; ojinjide@uic.edu (N.O.H.); bazil.labomascus@gmail.com (B.L.); amcleo2@uic.edu (A.M.)

**Keywords:** iron absorption, iron status, probiotic, human

## Abstract

Background: Strategies to prevent iron deficiency anemia (IDA) have varying effectiveness. The purpose of this systematic review of the literature and meta-analysis was to examine the effects of probiotics on iron absorption and iron status-related markers in humans. Methods: We followed the preferred reporting items for systematic reviews and meta-analyses (PRISMA) reporting guidelines. Relevant articles were identified from Embase, Pubmed, Scopus, and CINAHL from inception to February, 2019. We conducted a meta-analysis for eight studies examining the effect of the probiotic *Lactobacillus plantarum 299v (Lp299v)* on iron absorption. Results: Fifteen studies reported in 12 articles were identified (N = 950). Our meta-analysis of eight studies using a random-effects model demonstrated a significant increase in iron absorption following administration of the probiotic *Lp299v* with a pooled standardized mean difference (an average intervention effect size) of 0.55 (95% CI 0.22–0.88, *p* = 0.001). Of the seven randomized clinical trials (RCTs) and nonrandomized clinical trials examining a range of probiotic species on iron status, only one study supplementing with *Lp299v* showed improvement in serum iron; no other studies reported improvement in iron status-related indices with probiotic treatment. Conclusions: *Lp299v* significantly improved iron absorption in humans. Future research should include the assessment of *Lp299v* effect on iron absorption and iron status in populations at high risk of IDA, including pregnant women.

## 1. Introduction

Approximately half of all cases of anemia are due to iron deficiency anemia (IDA), affecting an estimated 136 million children, 16 million pregnant women, and 248 million non-pregnant women [1]. IDA has adverse short- and long-term health consequences, including impairments to cognition and physical development, severe fatigue, and reduced work capacity [2,3,4]. In 2016, an estimated 24,000 deaths globally were due to IDA, an increase of 33% since 2000 [5].

Optimizing iron status can be challenging. Currently, iron supplementation is a standard intervention for IDA. Barriers to body iron repletion via oral supplementation include underlying systemic inflammation from chronic conditions such as obesity [6], dietary factors such as phytates [7], and patient-reported symptoms including gastrointestinal distress [8]. The gut microbiota may also play an important role in iron bioavailability. Zimmermann and colleagues reported that oral iron supplements affect the composition of the gut microbiota, skewing it toward a more pro-inflammatory milieu that may contribute to decreased iron bioavailability [9]. Whereas the prebiotic supplement galacto-oligosaccharide selectively used by commensal *Bifidobacterium* spp. has been shown to enhance dietary iron absorption [10]. The study team reported gut microbiota composition (increased *Bifidobacterium* spp.) and microbial metabolic changes (i.e., increased short-chain fatty acid production) linked to decreased fecal pH likely played a role in iron enhancement. Fermented foods (e.g., yogurt, vegetables) have also been shown to enhance dietary iron absorption [11,12]. Lactic acid-forming bacteria, including lactobacilli, are thought to increase dietary iron bioavailability through several mechanisms such as reducing intestinal pH [13], shifts in gut microbiota metabolism and metabolite formation [13,14,15], and promotion of anti-inflammatory immunomodulation [16]. This suggests that probiotic bacteria may be a clinical tool to optimize dietary iron bioavailability to improve iron status without the gastrointestinal burden of additional supplemental iron.

Probiotics are defined as “live microorganisms that when administered in adequate amounts confer a health benefit on the host” by the International Scientific Association for Probiotics and Prebiotics [17]. Probiotics have gained public popularity because of their possible preventative and therapeutic effects, relative low cost, and accessibility. Probiotics improve iron absorption [12,13,18,19,20], but less certain is the effect of probiotics on body iron status [11,21,22,23,24,25]. The primary purpose of this systematic review of the literature was to comprehensively examine the existing evidence regarding the effects of probiotics on iron absorption and iron status in humans. The secondary purpose was to conduct a meta-analysis to examine the effect of the probiotic *Lactobacillus plantarum 299v (Lp299v)* on iron absorption in humans. The meta-analysis focused on *Lp299v* because there were a sufficient number of studies examining *Lp299v* on the same outcome, iron absorption, to conduct a meta-analysis.

## 2. Materials and Methods

We conducted this systematic review following the preferred reporting items for systematic reviews and meta-analyses (PRISMA) reporting guidelines [26].

### 2.1. Search Strategy and Study Selection

Using an a priori research protocol, relevant articles in English were identified from Embase, Pubmed, Scopus, and CINAHL from inception to February 2019 in consultation with a senior research librarian. The bibliographies of identified articles were also examined in an ancestry search. To conduct a systematic review evaluating all studies exploring the effect of probiotics on iron status and iron absorption in human subjects, we did not limit the search by study type. The search terms were organized by database and include both MeSH and Keyword searches.

(1) In the Embase database, the search term combinations used included ‘probiotic agent’/exp AND ‘iron’ exp, ‘probiotic agent’/exp AND ‘anemia’/exp, ‘probiotic agent’/exp AND ‘iron absorption’/exp, ‘anemia’/exp AND ‘bifidobacterium’/exp, ‘anemia’/exp AND ‘lactobacillus’/exp, ‘iron absorption’/exp AND ‘bifidobacterium’/exp, ‘iron absorption’/exp AND ‘lactobacillus’/exp. Search engine filters included article, human, and February 2019.

(2) In CINAHL, the search began with the MeSH headings: (MH “Probiotics”) AND (MH “Iron”), (MH “Probiotics”) AND (MH “Anemia”), (MH “Probiotics”) AND (MH “Iron”) AND (MH “Absorption”), (MH “Anemia”) AND (MH “Bifidobacterium”), (MH “Anemia”) AND (MH “Lactobacillus”). Keyword searches include anemia AND bifidobac, iron absorption AND bifidobac, probiotic AND anemia, probiotics AND iron, probiotics AND iron absorption, anemia AND bifidobacterium, anemia AND lactobacillus, iron absorption AND bifidobacterium, iron absorption AND lactobacillus. Search engine filters included academic journal, humans and February 2019.

(3) In Scopus, the Keyword search term combinations included probiotic AND iron, probiotics AND iron, probiotic AND anemia, probiotics AND anemia, probiotic AND iron absorption, probiotics AND iron absorption, anemia AND bifidobacterium, anemia AND bifidobacteria, anemia AND lactobacillus, anemia AND lactobacilli, iron absorption AND bifidobacterium, iron absorption AND bifidobacteria, iron absorption AND lactobacillus, iron absorption AND lactobacilli. Search engine filters included article, English, and February 2019.

(4) In Pubmed, the MeSH search terms included probiotic AND iron, probiotic AND anemia, probiotic AND iron absorption, anemia AND bifidobacterium, iron absorption AND lactobacillus, anemia AND lactobacillus, anemia AND bifidobac *, iron absorption AND bifidobacterium, iron absorption AND bifidobac *. Search engine filters included English and February 2019.

### 2.2. Eligibility Criteria

The criteria for article screening was determined a priori, with a focus on identification of journal articles that assessed the association between probiotics and iron status and iron absorption in human subjects. Following the database searches, two reviewers (N.O.H and B.L.) screened abstracts and titles to identify articles that fit the criteria for further review. Articles were included if they reported the effect of the exposure of interest (i.e., probiotics) on the outcomes of interest (iron status and iron absorption) in humans. The exposure of interest includes, but was not limited, to oral supplementation with probiotics including Lactobacillus and Bifidobacterium. The outcome of interest is iron-related biomarkers in the form of hemoglobin (Hb), hematocrit (Hct), serum iron, ferritin, serum transferrin receptor (sTfR), total iron-binding capacity (TIBC), and iron absorption of an iron isotope. Two independent reviewers (N.O.H and B.L.) searched for additional articles by reviewing the references of articles selected from the abstract and title searches. Only articles available in English were included in the search. Once articles were identified for potential inclusion through an abstract and title search, two different independent reviewers (M.D.K. and S.C.V.) fully reviewed the selected articles and selected the final articles for the systematic review. Disagreements were resolved through discussion and consensus among all authors.

### 2.3. Data Extraction and Management

A data abstraction form was created to collect key information including study design, study population, probiotics and iron compound used, colony forming units (CFU), intervention delivery method (e.g., capsule, drink), primary and secondary outcomes measured, and statistical analysis. Articles were organized by study design, randomized control trial or observational study, and outcome of interest (i.e., iron status and iron absorption). Two reviewers (M.D.K. and S.C.V.) extracted information from each selected article independently and compared findings. All discrepancies were resolved by discussion and consensus among all authors. Attempts were made to contact the authors of studies with unclear data.

### 2.4. Assessment Methodological Quality of Included Studies

Two reviewers (M.D.K. and S.C.V.) independently evaluated the methodologic quality of the selected studies using the Cochrane Review Bias Assessment Criteria for Intervention Studies [27] and the Cochrane Quality Assessment for Cross-Over Studies [28]. Randomized clinical trials (RCTs) were assessed based on several sources of bias: use of sequence generation, allocation concealment, blinding of participants and personnel, blinding of outcome assessors, completeness of outcome data, nonselective outcome reporting, and other measures of bias [27]. Cross-over studies were assessed based on similar criteria: appropriate cross-over design, randomized treatment order, carry-over effect, unbiased data, allocation concealment, blinding, incomplete outcome data, selective outcome reporting, and other bias [28]. For each criterion, studies were assessed as having a high risk of bias, low risk of bias, or an unclear risk of bias. Discrepancies between authors’ assessments of bias were discussed until consensus was reached.

### 2.5. Data Synthesis

Relevant data was extracted from selected studies and organized into a table format (Table 1).

### 2.6. Meta-Analysis

For the eight studies examining probiotic effects on iron absorption, we conducted a meta-analysis using means and standard deviations [30]. For two studies [12,19], we converted the 95% confidence interval (CI) to a standard deviation (SD) using the formula SD = sqrt (n) * (CI UL–CI LL)/t. For a third study [20], we initially converted standard error of the mean (SEM) SD = SE * sqrt (n), and then converted this to an SD. A meta-analysis of a subset of six studies that used a common measure of iron absorption was performed. This subset of studies adjusted the mean individual absorption ratio (test meal/reference dose) that was multiplied by 40 to obtain the percentage absorption of iron corresponding to a 40% reference dose absorption.

A random-effects model was used to calculate the standardized mean difference (SMD). Effect size heterogeneity was assessed using Q-statistics for testing the null hypothesis of homogeneity. Heterogeneity was also assessed through I^2^ and Tau^2^. Results were visualized using forest plots. In addition, we conducted a subgroup analysis excluding two studies [12,19], because the investigators did not adjust the absorption ratios to correspond to a reference dose absorption, as the other studies did. All analyses were conducted using Stata version 15 (StataCorp, College Station, TX, USA) using the user-developed “metan” command. Statistical significance was set at *p* < 0.05. Studies examining outcomes other than iron absorption were synthesized using a narrative analysis and not included in our meta-analyses because of heterogeneity in probiotics and iron status markers.

## 3. Results

### 3.1. Description of Selected Articles

**Figure 1:**
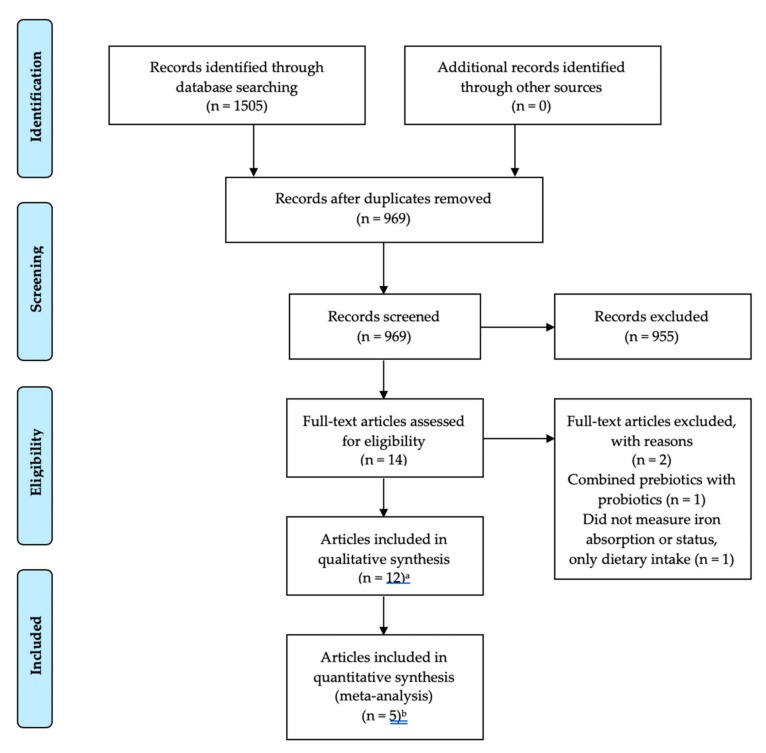
Flow chart for selection of studies. ^a^ 15 studies in 12 articles, ^b^ eight studies in five articles.

A total of 15 studies in 12 articles were identified using our search strategy of electronic databases. Figure 1 illustrates each stage of the selection process. Following the database search and subsequent review of titles and abstracts, 14 articles were initially considered for inclusion. After review of reference lists to identify other relevant articles and removal of articles not meeting inclusion criteria, a total of 12 articles met inclusion criteria. Three articles reported two trials for a total of 15 separate studies.

Characteristics of articles included are shown in Table 1. Five studies were RCT, two were two-group nonrandomized clinical trials, and eight used a cross-over design. Of the five RCT, three were double-blinded [21,25,29]. The nonrandomized clinical trials did not report blinding [23,24]. Of the eight cross-over studies, two were double-blinded [12,19], four studies were single-blinded [13,18], and two studies did not report blinding [20].

Sample sizes for all 15 studies ranged from 8 to 494, with half of the studies having less than 30 subjects (Table 1). The total sample size for only the eight studies included in the meta-analysis was 122 subjects. Across all of the studies, the two largest studies included children, 494 and 109, respectively [21,24], and another examined children with sleep problems and mild iron deficiency from the U.S. [29]. Seven study samples were healthy women of childbearing age [12,13,18,19,23]. One study included only pregnant women [11]. Another study included both healthy adult women and men [20]. Two studies included participants with older mean ages having a diagnosis: diabetes [11] and chronic low-dose aspirin users with unexplained iron deficiency anemia [22]. Most studies [12,13,18,19,20,22,29] included samples that are classified as high income based on the definition from The World Bank [31], followed by two studies which included samples classified as upper-middle income [23,24], and three studies with samples classified as lower-middle income [11,21,25].

Probiotic composition included *Lactobacillus casei* [21,22], *Lactobacillus reuteri* [21], *Lactobacillus acidophilus* [24], *Lp299v* [12,13,18,19,20,23,29], and multi-species cocktails [11,25] (Table 1).

There was wide variation in delivery of the probiotic, ranging from powder or capsule on an empty stomach [22,23] plus supplemental iron [29], capsule plus wheat buns made from fermented dough [13], capsule plus avoidance of fermented products [25], fruit drink with fermented oat base plus iron [18], milk between meals [24], milk plus coated straws [21], yogurt [11], oat gruel only [12], oat gruel and wheat roll with fermented oat base [19], bread rolls wheat flour (low phytate) or wheat bran (high phytate) plus fermented or fresh veggies [20] (Table 1). The addition of low phytate/high phytate meals was to determine whether the probiotic could overcome the effect of phytate on non-heme iron absorption. Some studies addressed potential bias related to dietary and nutrient supplements that might have influenced iron absorption and iron status markers through design and delivery methods so that all participants were exposed to the same delivery method or compared across products and delivery method. In the RCT by Rosen and colleagues [29] that included study participants with iron deficiency anemia, all participants received supplemental iron based on standard dosing.

Iron salt composition was reported in three studies [18,23,29]. Hoppe et al. (2015) [18] added ferrous lactate dehydrate to a fruit drink. Korcock et al. (2018) [23] added Sucrosomial^®^ iron, and Rosen et al. (2019) [29] added ferrous sulphate to a capsule.

Duration of probiotic use in RCT and nonrandomized clinical trials varied widely: 7 days [23], 6–8 weeks [29], 8 weeks [25], 9 weeks [11], 3 months [22], an estimated 5 months based on 101 school days [24], 6 months [21]. Across these study periods, participants took the probiotic once per day, except for Agustina et al. colleagues [21], who had participants take the probiotic twice per day.

Several outcomes were reported across studies: non-heme iron absorption [12,18,19,20], serum iron [11,23,24], serum ferritin [21,23,25,29], Hb [21,22,23,24], Hct [21,24], serum transferrin receptor (sTfR) [21], and total iron binding capacity (TIBC) [23].

### 3.2. Bias

**Figure 2:**
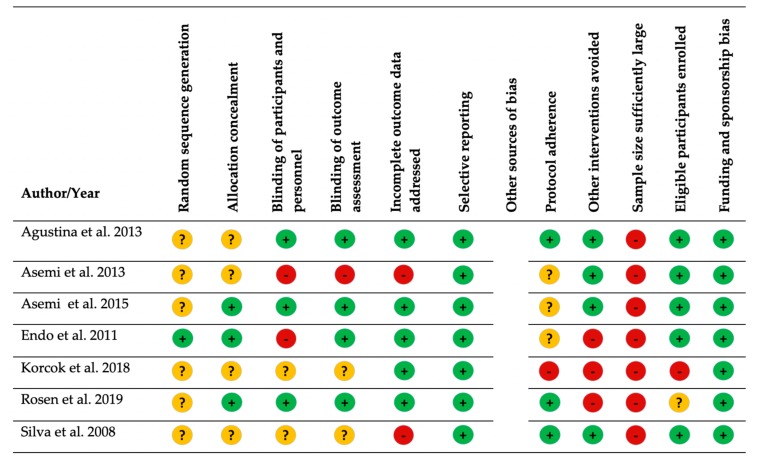
Bias assessment of intervention studies with comparison groups. Green circle with plus sign = low risk of bias; yellow circle with question mark = unclear risk of bias; red circle with minus sign = high risk of bias.

**Figure 3:**
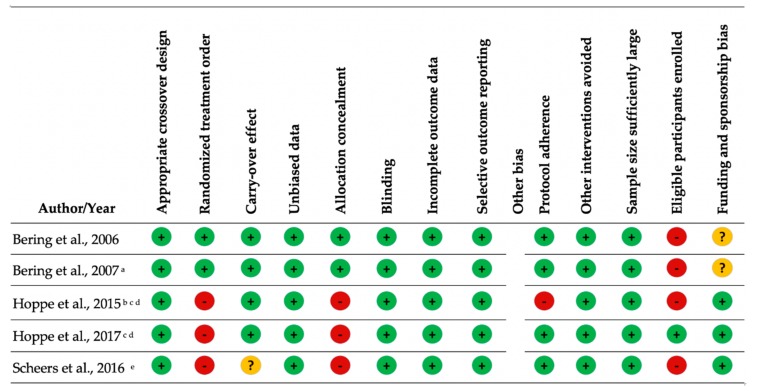
Quality assessment for crossover studies. Green circle with plus sign = low risk of bias; yellow circle with question mark = unclear risk of bias; red circle with minus sign = high risk of bias ^a^ Carry-over controlled for in the analysis. Results do not report carry-over effect. ^b^ Reports two studies in one article; same methodology except tested different colony forming units (CFU) per gram of *L. plantarum 299v*. ^c^ Placebo administered before probiotic. ^d^ Reports two studies in one article; same methodology. ^e^ Reports two studies in one article; same methodology except tested subjects with low or high phytate bread rolls.

The methodological quality of the studies varied. Overall, there was less bias in the cross-over studies compared with the RCTs and nonrandomized clinical trials (Figure 2 and Figure 3). Key sources of bias in the RCTs and nonrandomized clinical trials included (1) whether the sample size was sufficiently large to detect a change in iron status [11,21,22,23,24,25,29] and (2) whether other interventions (probiotic products, iron supplementation, and dietary sources of phytate) were avoided [22,23,29]. Key sources of bias in the cross-over studies included: (1) a lack of description on whether all eligible participants that met prespecified criteria were enrolled, and (2) blinding was either single-blind [13,18] or not reported [20] (Supplementary File).

### 3.3. Iron Absorption

**Figure 4:**
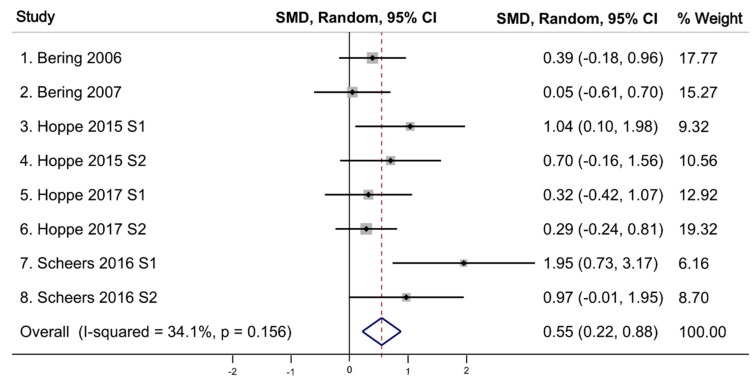
Effect of probiotic (*Lp299v*) on iron absorption for all eight studies. SMD: standardized mean difference; CI: confidence interval; I-squared: variation in SMD attributable to heterogeneity; S: study; *p* value associated with Q statistic.

**Figure 5:**
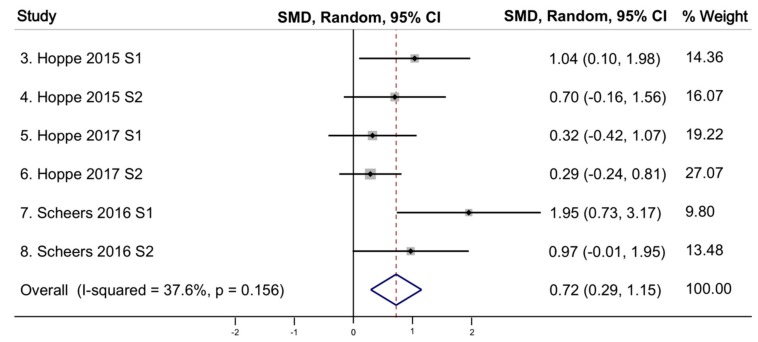
Effect of probiotic (*Lp299v*) on iron absorption for subgroup analysis of six studies. SMD: standardized mean difference; CI: confidence interval; I-squared: variation in SMD attributable to heterogeneity; S: study; *p* value associated with Q statistic.

All eight studies (reported in five articles) that examined the effect of the probiotic *Lp299v* on iron absorption [12,13,18,19,20] independently found a statistically significant increase in iron absorption among those ingesting *Lp299v*. All these studies used a cross-over design. Table 2 shows the comparison of the meta-analytic effects, represented by pooled standardized mean difference (SMD), the level of variation around the mean (Q-statistic), and study heterogeneity (Tau^2^). Our meta-analysis of all eight studies using a random-effects model demonstrated a significant increase in iron absorption following administration of the probiotic *Lp299v* with a pooled SMD (an average intervention effect size) of 0.55 (95% CI 0.22–0.88, *p* = 0.001) (Figure 4). The heterogeneity test using the Q-static of 10.62 was not significant, indicating an absence of heterogeneity of effect size across studies. Homogeneity of these eight studies was observed with a T^2^ of 0.0747, indicating the studies are statistically homogenous. Analyses of these eight studies using fixed-effects model also found a statistically significant increase in iron absorption following administration of the probiotic *Lp299v* (pooled SMD = 0.49), and a Q-statistic and T^2^ indicating studies are statistically homogeneous (*p*-value = 0.156). Finally, we conducted a sub-analysis excluding the two Bering studies [12,19] that did not adjust the mean absorption ratio corresponding to a reference dose absorption and found the effect size was larger with the random effects (pooled SMD = 0.72) (Figure 5) [13,18,20]. The Q-statistic and T^2^ continued to support statistical homogeneity across studies.

### 3.4. Iron Status Markers and Hemoglobin

Several iron status-related outcomes were reported across studies, including serum iron, ferritin, Hb, Hct, sTfR, and TIBC. Four studies examined serum iron [11,23,24,25], four studies investigated ferritin [21,23,24,29], two studies examined only Hb [22,23], and two studies examined Hb and Hct [21,24], one study examined sTfR [21], and one study examined TIBC [23]. Only one study found an improvement in serum iron in the probiotic arm, and the species tested was *Lp299v* [23]. No significant differences were found between probiotic and control groups for any of these markers. Endo et al. [22] reported a significant increase in serum iron within the *L. casei* group, but did not find a statistical difference between the probiotic and placebo groups.

## 4. Discussion

This is the first systematic review and meta-analysis to our knowledge to examine the effect of probiotics on iron absorption and iron status in humans. Overall, the probiotic *Lp299v* significantly increased non-heme dietary iron absorption in crossover-designed studies compared with a control period. The standard mean difference detected between control and test (probiotic) in our meta-analysis was 0.55, indicating a moderate effect size consistent with iron absorption enhancement. It is suggested that *Lp299v* confers a beneficial effect on dietary non-heme iron absorption through several mechanisms including: (1) microbial metabolite production of p-hydroxyphenyllactic acid [15], a microbial by-product that can promote the reduction of ferric iron to the more bioavailable ferrous form [13]; (2) enhanced mucin production at the intestinal surface, promoting enterocyte iron uptake [14]; and (3) immunomodulation, promoting an anti-inflammatory immune response [16] that suppresses hepcidin [13], the master regulator of systemic iron homeostasis [32], enhancing iron bioavailability.

In our analysis, only one study reported an increase in serum iron in probiotic (*Lp299v*) supplemented subjects vs. controls [23]. The lack of significant improvement in iron status or Hb in the other six studies examining effects on iron status may be due to wide variation across the studies in probiotic species, dosage, and duration of use of the probiotic.

There may have been several factors that led to enhanced iron absorption with probiotic treatment, yet results were equivocal for improvements in iron status. First, none of the RCTs and nonrandomized clinical trials were powered to detect a difference in iron status. In contrast, the absorption studies were powered adequately for the main outcome of iron absorption. This likely impacted the ability to detect a significant effect of probiotic supplementation on iron status outcomes. The baseline iron status may have differed in the iron absorption studies versus the studies examining longer-term effects on body iron status. The baseline ferritin of participants in the absorption studies was relatively low, ranging from 12 to 33 ng/dL [12,13,18,19]. Iron absorption is significantly enhanced in most persons with depleted iron stores. In the only clinical trial that reported iron status indicators at baseline, mean ferritin was low [24]. It is possible that the baseline iron status was adequate in the other clinical trials, thus a significant improvement in body iron status would not be anticipated. Finally, adherence to the probiotic intervention was not consistently reported among the clinical trials, with one study not monitoring adherence [23], and several other studies not describing the rates of adherence [11,22,25]. Low adherence to the probiotic interventions could contribute to the observed null effects on body iron status.

More than half the studies were conducted with homogenous samples. All the studies that found *Lp299v* effective at enhancing iron absorption included primarily healthy white European women from high-income countries [31]. The one study showing probiotic supplementation increased serum iron concentrations was also conducted with healthy white females [23]. This homogeneity limits the generalizability of the study findings. Further trials with more diverse samples are needed to assess the robustness of the effect of *Lp299v* on iron absorption and to examine long-term effects on iron status markers. Specifically, studies investigating populations at greatest risk of iron deficiency, including pregnant women are needed.

Only three studies reported the iron salt used. Therefore, it is not known whether iron salts impacted the results of both the iron absorption- and iron status-related studies. The most commonly prescribed iron supplements for iron repletion are ferrous salts, including ferrous sulphate and ferrous fumarate. However, the estimated absorption rate of ferrous salts is 10–15% [33]. Sucrosomial^®^ iron, a newer iron formulation in which ferric pyrophosphate is protected by a phospholipid bilayer and sucrosome, has been shown to be better tolerated than oral iron salts and has demonstrated high iron bioavailability [34]. Korcock et al. (2018) [23] reported using Sucrosomial^®^ iron and was the only study that found an increase in serum iron between the probiotic and control groups. Moving forward, studies should report the type of iron salt used.

The RCT and nonrandomized trials studies had methodological challenges that likely affected their findings. First, the RCT and nonrandomized trials did not have adequate statistical power to detect differences between study groups, except for Korcok and colleagues [23]. Many of the studies were powered on outcomes other than iron status, potentially contributing to the lack of effect [11,21,22]. Second, there were variations in adherence to the probiotic intervention, reporting of adherence, and avoidance of other interventions, such as sources of iron and dietary inhibitors of iron absorption. Third, many studies did not include a comprehensive assessment of baseline iron status, including markers of total iron, ferritin, and Hb concentration, along with hepcidin, erythropoiesis, and underlying systemic inflammation, all known to affect iron bioavailability and iron status [32]. For example, in the presence of systemic inflammation, hepcidin increases, resulting in diminished expression of the ferroportin-1 iron exporter, ultimately reducing iron flow from stores and diet. Together, these methodological challenges might have reduced the ability of the trials to detect the effects of the probiotic on iron status. Future studies need to better monitor and report adherence to the probiotic intervention and include the collection of other lifestyle factors such as diet, and other parameters such as circulating markers of inflammation, a more comprehensive assessment of baseline iron status, and hepcidin to accurately determine the effect of probiotic supplementation on iron homeostasis.

A limitation of this review is that we included studies that examined both single and multispecies probiotics, and studies that examined iron status as a secondary outcome without a priori knowledge of the statistical power to detect an effect. We included these studies because there are a limited number of human studies on the effect of probiotics on iron absorption and status. Our initial meta-analysis of eight studies included two that used a different measurement approach to assess iron absorption. To address this limitation, we conducted a sub-analysis of the six studies using the same measurement approach. The robustness of our meta-analysis is strengthened because all the absorption studies used the same probiotic, *Lp299v.*

## 5. Conclusions

Our meta-analysis of eight studies found that *Lp299v* increased dietary non-heme iron absorption in healthy white Europeans who were primarily women. The potential effect of probiotics other than *Lp299v* on iron absorption is unknown. Out of the seven studies that measured iron status, only one study reported a positive effect of *Lp299v* on serum iron. None of the other probiotics had a significant effect on iron status. Future studies should test the long-term effect of *Lp299v* on iron status in vulnerable populations, including pregnant women. These studies need to include a comprehensive assessment of iron status and other important lifestyle and biochemical markers of inflammation and iron metabolism that affect iron homeostasis.

## Figures and Tables

**Table 1:** Study characteristics.

Study/Year	Subjects	Baseline Iron Status	Design/ Blinding	Study Groups or Product	Iron Compound	Intervention Delivery Method, Frequency and Duration	Effect on Iron Status or Iron Absorption	Included in Meta-Analysis
**RCTs and Nonrandomized Clinical Trials**								
Agustina et al., 2013 [21]	Indonesia N = 494 Children Age: 1–6 Healthy Non-breastfed	IDA n (%) Group 1: 12 (10) Group 2: 17 (14) Group 3: 13 (11) Group 4: 14 (11)	RCT double-blinded	Group 1: Placebo–Low calcium, ~50 mg/d Group 2: Placebo–Regular calcium, ~440 mg/d Group 3: *L. casei 431* 1: 5 × 10^8^ CFU/d plus regular calcium Group 4: *L. reuteri* 17,938 5 × 10^8^ CFU/d plus regular calcium	Not reported	180 mL low-lactose milk and coated straws Twice daily across 6 months	↔ Hb ↔ Hct ↔ serum ferritin↔ sTfR	No
Asemi et al., 2013 [11]	Iran N = 70 Pregnant, third trimester Age: 18–30	Iron (mg/dL) Group 1: 124 ± 88.3 Group 2: 118 ± 61.8	RCT single-blinded	Group 1: Placebo Group 2: *L. acidophilus* 1 × 10^7^ CFU, *B. lactis* 1 × 10^7^ CFU	Not reported	200 g yogurt Daily across 9 weeks	↔ serum iron	No
Asemi et al., 2015 [25]	Iran N = 58 Diabetic patients, not pregnant Age: 52.1 ± 6.9 (Group 1) 49.6 ± 9.9 (Group 2) Female % not reported	Iron (mg/dL) Group 1: 66.1 ± 33 Group 2: 69.5 ± 62.9	RCT double-blinded	Group 1: Placebo Group 2: *L. acidophilus* 2 × 10^9^ CFU, *L. casei* 7 × 10^9^ CFU, *L. bulgaricus* 2 × 10^8^ CFU, *B. breve* 2 × 10^8^ CFU, *B. longum* 7 × 10^9^ CFU, *S. thermophiles* 1.5 × 10^9^ CFU	Not reported	Capsule Daily across 8 weeks	↔ serum iron	No
Endo et al., 2011 [22]	Japan N = 25 Female 28% Age: 70.3 ± 6.2 (Group 1) 73.9 ± 8.5 (Group 2) Unexplained IDA on chronic low-dose aspirin	Hb (g/dl) Group 1: 10.9 ± 1.7 Group 2: 10.2 ± 2	RCT single-blinded	Group 1: No placebo Group 2: *L. casei* 45 × 10^8^–63 × 10 CFU	Not reported	Powder Daily for 3 months	↔ Hb (between groups) ↑ Hb (within *L. casei* group)	No
Korcok et al., 2018 [23]	Serbia N = 20 Female 100% Healthy	Not reported	Two group comparison	Group 1: Placebo Group 2: *L. plantarum 299v* 1.1 × 10^9^ CFU	Sucrosomal iron 10 mg	Capsule plus supplemental iron and 15 mg vitamin C 7 consecutive mornings Empty stomach	↑ serum iron ^a^↔ serum ferritin ↔ TIBC ↔ Hb	No
Rosen et al., 2019 [29]	United States N = 52 Children with mild iron deficiency (ferritin < 50 ng/mL), and insomnia or restless sleep, 58% had psychiatric and/or mental health diagnosis Age: 5–18	Not reported	RCT Double-blinded	Group 1: placebo Group 2: *L. plantarum 299v* 1.7 × 10^10^ CFU	Ferrous sulphate 325 mg or ferrous sulphate 15 mg elemental iron/mL (if children < 20 kg, dose of 3 mg/kg/d up to maximum dose 65 mg; in children > 20 kg range 0.4–3 mg/kg/d)	Capsule plus supplemental iron and vitamin C (125 mg < 5 years of age and 250 mg > 5 years of age) 6–8 weeks No milk or food within 2 h of medication	↔ serum ferritin	No
Silva et al., 2008 [24]	Brazil N = 109 Children Age: 20–62 months	Iron (µg/dL) Group 1: 48.9 ± 9.8 Group 2: 49 ± 10.5 Hb (g/dL) Group 1: 12.2 ± 0.7 G 2: 12.1 ± 0.7 Ferritin (ng/mL) Group 1: 41.2 ± 21.4 Group 2: 38.5 ± 19	Two group comparison	Group 1: Placebo Group 2: *L. acidophilus* 10^8^ CFU	Not reported	80 mL whole milk beverage with 3 mg iron 1.2 g culture of *L. acidophilus* Daily between lunch and afternoon snack Monday to Friday during 101 class days	Between groups ↔ Hb Within *L. acidophilus* group ↓ Hb ↓ Hct ↓ serum iron ↓ serum ferritin Within Placebo group ↓ Hb ↓ Hct ↔ serum iron ↑ serum ferritin	No
**Cross-over Studies**								
Bering et al., 2006 [12]	Denmark N = 24 Female 100% Age: 25 ± 4 low iron stores not anemic, not pregnant, not lactating	Hb (g/L) Range: 111–137 Ferritin (µg/L) Range 12–40	Cross-over double-blinded	Product A: *L. plantarum 299v* 1.1 × 10^9^ CFU, fermented gruel Product B: pasteurized Product A Product C: non-fermented gruel (pH adjusted with lactic acid) Product D: control meal non-fermented gruel with added organic acids (lactic acid and acetic acid)	Not reported	100 g oat gruel Product A, B & D: non-heme Fe 2.8 mg Product C: non-heme Fe 2.5 mg Twice on four consecutive mornings 12 h of fasting	↑ non-heme iron absorption	Yes
Bering et al., 2007 [19]	Denmark N = 18 Female 100% Age: 22 ± 3 low iron stores not anemic, not pregnant, not lactating	Hb (g/L) Range: 116–135 Ferritin (µg/L) Range 13–29	Cross-over double-blinded	Product A: heat-inactivated lactic acid gruel Product B: viable lypholized *L. Plantarum 299v* 1.1 × 10^9^ CFU, heat-inactivated lactic acid gruel	Not reported	100 g fermented, pasteurized oat gruel plus 140 g whole-wheat roll Product A & B: non-heme Fe 1.9 mg Twice on two consecutive mornings12 h of fasting	↔ non-heme iron absorption	Yes
Hoppe et al., 2015 [18]	Sweden N = 10 (Study 1) N = 11 (Study 2) Female 100% Healthy Age: 24.3, range 20–40 (Total sample)	Iron (µmol/L) Study 1: 15 ± 6 Study 2: 18 ± 7 Hb (g/L) Study 1: 138 ± 8 Study 2: 135 ± 9 Ferritin (µg/L) Study 1: 33 ± 13 Study 2: 33 ± 14	Cross-over single-blinded	Product A: Placebo Product B: *L. plantarum 299v* 1.3 × 10^9^ CFU (Study 1) *L. plantarum 299v* 1.7 × 10^10^ CFU (Study 2)	Ferrous lactate dehydrate 4.2 mg	200 mL fruit drink with fermented oat base plus iron (2.1 mg/100 mL) Product A: non-heme Fe 5.2 mg (Study 1) non-heme Fe 5.4 mg (Study 2) Product B: non-heme Fe 4.6 mg (Study 1) non-heme Fe 5.2 mg (Study 2) 4 consecutive days Empty stomach	↑ non-heme iron absorption (Study 1) ↔ non-heme iron absorption (Study 2)	Yes
Hoppe et al., 2017 [13]	Sweden N = 14 (Study 1) N = 28 (Study 2) Female 100% Age: 26.2 ± 4.6 (Study 1) 25.6 ± 6.8 (Study 2) Healthy	Iron (µmol/L) Study 1: 15 ± 5 Study 2: 16 ± 7 Hb (g/L) Study 1: 135 ± 6 Study 2: 134 ± 10 Ferritin (µg/L) Study 1: 30 ± 21 Study 2: 27 ± 14	Cross-over single-blinded	Product A: Placebo Product B: *L. plantarum 299v* 10^10^ CFU (Study 1 & 2)	Not reported	Capsule plus two wheat breakfast buns made with fermented dough Product A & B: non-heme Fe 4.2 mg 4 consecutive days Empty stomach	↑ non-heme iron absorption (Study 1) ↑ non-heme iron absorption (Study 2)	Yes
Scheers et al., 2016 [20]	Sweden N = 8 low-phytate (Study 1) N = 9 high-phytate (Study 2) Female 35% Age: 21–54 Healthy	Not reported	Cross-over	Product A: Fresh vegetables Product B: *L. plantarum 299v* 2.4 × 10^9^ CFU fermented vegetables	Not reported	140 g bread rolls (low phytate had wheat flour; or high phytate had wheat bran and wheat flour) plus 100 g fermented or fresh vegetables Low phytate non-heme Fe 4.4 mg High phytate non-heme Fe 4.0 mg Alternate mornings on 4 consecutive days Overnight fast	↑ non-heme iron absorption	Yes

L. = lactobacillus, B. = bifidobacterium, S. = streptococcus. **↑** statistically significant increase, ↓ statistically significant decrease, ↔ no statistically significant difference. IDA refers to iron deficiency anemia, RCT refers to randomized clinical trial, CFU refers to colony forming units, Fe refers to iron, Hb refers to hemoglobin, Hct refers to hematocrit, TIBC refers to total iron binding capacity, sTfR refers to serum transferrin receptor. ^a^ Statistical analysis and significance not reported.

**Table 2:** Effect of probiotic on iron absorption.

Number of Studies	Model	Effect: Pooled SMD	95% CI	*p*-Value	Q	*p*-Value	Tau Squared
8	random	0.55	0.22, 0.88	0.001	10.62	0.156	0.0747
6 ^a^	random	0.72	0.29, 1.15	0.001	8.01	0.156	0.1058

^a^ Excludes Bering et al. [12,19] studies; includes only studies that adjusted the mean individual absorption ratio (test meal/reference dose) that was multiplied by 40 to obtain the percentage absorption of iron corresponding to a 40% reference dose absorption.

## Supplementary Materials

The following are available online at https://www.mdpi.com/2072-6643/11/12/2938/s1.
