# Supplementary material for: A Systematic Review and Meta-Analysis on the Effects of Probiotic Species on Iron Absorption and Iron Status
Source: Nutrients. 2019 Dec 3;11(12):2938. doi: 10.3390/nu11122938 (PMC6949908; doi:10.3390/nu11122938)
Supplement: Supplementary file 1 [file nutrients-11-02938-s001.pdf]

The methodological quality of the studies varied. Key sources of bias included: (Figures 2 and 3).

1. *Random sequence generation* (selection bias). The method of randomization was not adequately described in four of the five RCT studies[14, 24, 28, 33] and two studies were non-randomized clinical trials[26, 27] therefore the risk of bias is high. Only one RCT was at low risk for this bias[25]. This bias is not applicable for the cross-over designs.
2. *Allocation concealment* (selection bias). The method used to conceal the allocation sequence was not described in sufficient detail in four of the seven RCT and non-randomized clinical trials[14, 24, 26, 27] and in six of the eight studies using cross-over designs reported in three articles[16, 21, 23].
3. *Blinding of participants and personnel* (performance bias). Two of the seven RCT and non-randomized clinical trials[26, 27] did not describe measures used to blind study participants and personnel from knowledge of which intervention a participant received, one RCT[14] did not blind personnel; and one RCT did not blind participants[25]. Two cross-over studies reported by Scheers and colleagues [23] delivered the probiotic in fermented vegetables while the comparison meal was fresh vegetables; however, this lack of blinding did not likely affect the outcome assessment. Other cross-over studies used an adequate blinding method to prevent performance bias[15, 16, 21, 22].
4. *Blinding of outcome assessment* (detection bias). There was low risk of detection bias across most studies. Two out of seven RCT and non-randomized clinical trials were single blind[14, 25] and two trails did not describe their blinding method[26, 27], so the risk of bias is unclear. All cross-over studies used an adequate blinding method to assess outcomes and prevent detection bias[15, 16, 21-23].
5. *Incomplete outcome data addressed* (attrition bias). Two of the seven RCT and non-randomized clinical trials[14, 27] did not describe the completeness of outcome data for each main outcome, including attrition and exclusions from the analysis. All the cross-over studies had no missing outcome data or the reason for missing data was described[15, 16, 21-23].
6. *Selective reporting* (reporting bias). All the RCT and non-randomized clinical trials and cross-over design studies were low risk for reporting bias because investigators fully reported their outcomes.
7. *Appropriate cross-over design*. All of the studies using a cross-over design had low risk of bias based the appropriate use of this design: 1) the condition of the subjects were chronic or stable, 2) the probiotic was intended to produce a temporary change and 3) the effect of the first intervention period did not last into the second intervention treatment period or any residual effect was taken into account.
8. *Randomization of treatment order in cross-over designs.* In four of the eight studies, the placebo was always administered first[16, 21], and in two studies randomization of treatment order was not reported[23]. Two studies randomized treatment order[15, 22].
9. *Carry-over effect in cross-over designs.* Two studies varied the order of the interventions and did not discuss a carry-over effect[23]. Two studies controlled for the carry-over effect in the analysis and had wash-out periods[15, 22] and four studies did not have carry-over bias because the placebo was administered first[16, 21].
10. *Other bias*

a. *Protocol adherence.* Two studies were rated as high for bias related to protocol adherence[21, 26]. Korcok et. al.[26], did not report monitoring of or rates of adherence. Hoppe et al.[21] reported poor adherence to fasting for both the study meals and reference iron dose, however adherence rates to fasting were not reported. Three studies were rated as having unclear bias[14, 25, 28]. Asemi et al.[14, 28] reported adherence monitoring methods, including phone calls and dietary logs, but did not report adherence rates. Endo et al.[25],reported one subject was dropped from

the analysis because of poor compliance, but how adherence was measured across the three-month study period was not described.

b. *Other interventions avoided*. Three of the 15 studies were at risk for bias related to exposure to other interventions that could impact the outcomes, specifically other probiotic products, iron supplementation, and dietary sources of phytate that might affect iron absorption[25, 26, 33]. Although Endo et al.[25], excluded subjects who took probiotics three months prior to study, they did not collect dietary records during the trial to capture whether the participants were consuming other probiotic products and/or monitoring other sources of dietary or supplemental iron, and phytate during the study. Korcok et al.[26], and Rosen et al.[33] did not state inclusion or exclusion criteria related to probiotic consumption, nor whether other interventions were avoided during the course of the study. Asemi[14, 28], conducted a 2-week run-in period with no consumption of probiotic products before randomization.

c. *Sample size sufficiently large*. All the RCTs and non-randomized clinical trials were rated as high risk for bias due to the sample not being sufficiently large enough to detect a difference in iron status between the intervention and control groups[14, 24-27, 33] . One RCT was powered on the frequency and duration of diarrheal episodes[24]. Two studies were powered on serum alanine aminotransferase levels[14, 28]. The non-randomized clinical trials[26, 27] and one RCT[33] did not include a description of power. Power was sufficient to detect a change in iron absorption in all cross-over studies[15, 16, 21-23].

d. *Eligible participants enrolled.* One non-randomized clinical trial[26] and four of five cross-over studies[15, 21-23] were rated as being at high risk because they did not describe whether all eligible participants that met prespecified entry criteria were enrolled. One RCT[33] did not specify the number of potential subjects who did not meet inclusion criteria versus declined participation, therefore the risk of bias is unclear.

e. *Funding and sponsorship bias.* All the RCTs and non-randomized clinical trials were rated as having a low risk for bias from funding and/or sponsorship influence. Two of the eight cross-over studies were rated as unclear because a co-author was also an employee of the company that supplied the probiotic, there was no description of this author's role on the study, and there was no statement describing any conflict of interest[15, 22]. Although, the risk of funding and sponsorship bias was rated as low, three studies had potential conflicts of interests: either one or two authors[16, 21] were employees of the probiotic company (ProbiAB) that supplied the product for the studies. The contributions of these authors were described, and a conflict of interest statement was included. One RCT [24] had two authors who were employees of a global consumer goods company (Unilever); however, the probiotic was provided by a different manufacturer (BioGaia AB) and a no conflict of interest was reported. The other studies reported public funding, had authors who were not associated with a probiotic manufacturer, and/or had statements indicating no conflict of interest[14, 23, 25-27].
